# Cochleo-vestibular clinical findings among drug resistant Tuberculosis Patients on therapy-a pilot study

**DOI:** 10.1186/1755-7682-5-3

**Published:** 2012-01-31

**Authors:** Lebogang Ramma, Titus S Ibekwe

**Affiliations:** 1 Division of Communication Sciences & Disorders, Faculty of Health Sciences, University of Cape Town Groote Schuur Hospital, Old main building, F-45, Observatory, Cape Town, 7925, South Africa; 2 Department of ENT Surgery, College of Health Sciences, University of Abuja, PMB 117 Abuja Nigeria

**Keywords:** Multidrug resistance, Extreme drug resistance, Aminoglycosides, ototoxicity

## Abstract

**Abstracts:**

**Background**

To investigate the Cochleo-vestibular clinical and audiometric findings in Multi and Extreme Drug Resistance(MDR and XDR) tuberculosis(TB) patients on treatment and make recommendations.

**Methods**

A cross-sectional study of adult MDR and XDR-TB patients was conducted in a general hospital in Cape-Town-South-Africa. Ethical approval was secured and all consenting patients administered with pretested and validated questionnaire under the guidance of International Classification of Functioning, Disability and Health(ICF) Checklist-version-2.1a. Audiometric evaluation included: Otoscopy, Diagnostic Audiometry and Tympanometry. The data analyses were done with SPSS version 16, Chi-square and StatCalc-7.

**Results**

Fifty-three adults, ages 18-60 (mean-33 years) comprising 26 males and 27 females participated in the study. Hospital stay duration varied from 1-18 months (mean-6 months) and all were on anti-Koch's second line drugs (regimen 2). MDR TB group were 45(85%) and XDR 8(15%). Vertigo was the most common vestibular symptoms, 24(45%) whereas, tinnitus 23(42%) and hearing loss 13(25%) were most frequent auditory complaints. Bilateral sensorineural hearing losses of varying degrees were confirmed in 23(47%).

There was no association between gender and age with hearing loss [χ^2 ^(P = 0.16, ά = 0.05) and (p = 0.13, ά = 0.05)]. Furthermore, MDR and XTR TB groups [20/42 Vs 3/8; Z = 0.46 and P = 0.64], showed no difference in pattern of the hearing losses.

**Conclusions**

A multi-disciplinary close surveillance of MDR and XDR TB patients on therapy is imperative. Finally, researches into therapeutic trials on antidotes and potent safer substitutes for aminoglycosides in the management are recommended.

## Introduction

Following the advent of HIV-AIDS in 1981 there has been the explosion of opportunistic infections and global resurgence of diseases, prominent among which is tuberculosis (TB). The control of this disease especially in the developing world has suffered a great set back following resistance to conventional regimen for treatment of TB. As a result, the prevalence of multidrug-resistant (MDR) and extensively drug-resistant (XDR) tuberculosis particularly in Africa has been on the rise. Meyer and McAdam in 2007, reported an alarming rate of about to 1000/1000 000 TB infections including the resistant strains in the Western Cape Province of South Africa [1].

In South Africa, TB patients typically undergo treatment with first line TB treatment (regimen 1) drugs: Rifampicin, isoniazid (INH), pyrazinamide, and ethambutol [2]. MDR TB is resistance to at least two main first-line TB drugs (Isoniazid and Rifampicin) and XDR to three or more of the six classes of second-line drugs respectively [3]. To this end, patients are left with the option of management with extreme drugs like Amikacin, streptomycin belonging to the aminoglycosides, chemotherapeutic agents with potential ototoxicity and nephrotoxicity. Some of these side effects could be worse than the initial symptoms. Absolute loss of hearing loss, tinnitus, and vertiginous spells are among common cochlea-vestibular disorders that could arise from the treatment. This poses a huge potential threat to the eradication of the MDR and XDR TB since side effects could promote low compliance and high attrition rate to treatment.

Therefore, there is need for monitoring of the patients by health personnel during the course of management to curtail the side effects and encourage compliance to treatment.

It is following this that we conducted a survey among the in-patients undergoing MDR and XDR TB treatment in a general hospital in the province of Western Cape of South Africa to ascertain their spectrum of otovestibular clinical symptoms and audiometric profiles. It is hoped that this will act as a guide to the physicians involved in the management of this disease.

## Materials and methods

This was a cross sectional study involving adult patients (ages 18-60 years) on ward admission diagnosed and currently under treatment for MDR or XDR TB. The drugs were administered by Specialist Medical team at right dose and durations. Volunteers who were within this age range and on admission for treatment lasting at least 2 weeks were consecutively selected into this survey. The essence of the closed age selection was to ensure validity of consent (at least age 18) and to limit the effect of presbycusis (beyond age 60 years). Minimum period of admission of 2 weeks was to ensure enough compliance and exposure of patients to the drugs. Patients with evidence suggestive of hearing loss prior to the commencement of therapy were excluded from the study.

### Consent

Prior to the commencement of the study, ethical approval was obtained from the Human Research Ethic Committee of the Faculty of Health Sciences of the University of Cape Town. In addition, permission to conduct the research was also granted by the hospital management of the participating hospital and the Western Cape Provincial Department of Health. Call for participants were sourced and respondents were asked to indicate their willingness to take part in this study through the endorsement of consent forms.

### Instrument and data collection

Major instrument used for this work was a self developed questionnaire under the guidance of International Classification of Functioning, Disability and Health (ICF) - Checklist version 2.1a. Inputs were also made by 4 clinical audiologists with at least 5 years of experience (see Additional file 1). The questionnaire comprises three key sections: Demographic information, Impairment of Body Functions, and Activities limitations & Participation restrictions. The questionnaire was pretested on 12 patients in the same institution prior to the commencement of the study. The outcome of the pre-test was used for validation of the questions. Subsequently, a survey was conducted using the self administered and pre-tested/validated questionnaires.

Following the administration of the questionnaire, audiometric evaluations were conducted for the patients via otoscopy using Heine Mini 2000 Otoscope (to eliminate wax impaction and pre-existing auditory canal and tympanic membrane defects), GSI-38 screening tympanometry was also used (to rule out middle ear disorders) and Pure Tone Audiometry with model GSI 61 diagnostic audiometer in a standard sound proof boot(to ascertain the degree and type of hearing loss).

### Data analysis

The SPSS version 16, Chi-square and StatCalc-7 were used for data entrance, cleaning and analysis.

## Results

Fifty-three adults, ages 18-60 (mean 33 years) comprising 26 males and 27 females took part in the study. Their length of hospital stays varied from 1- 18 months (mean: 6 months). They were all on anti-koch's second line drugs treatment (i.e. regimen 2). Those on Multi-Drug Resistant (MDR) TB treatment were 45 (85%) and extensively drug-resistant (XDR) TB 8(15%). No previous established history of hearing loss prior to enrolment into the therapy was established from any of the participants, however; 15(29%) had history of noise exposure in previous jobs but no evidence of resultant hearing loss

**Figure 1:**
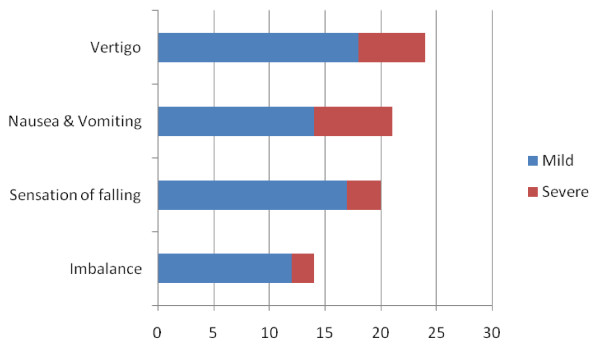
**Most frequently reported vestibular-balance problems**. The common vestibular-balance problems reported by the participants (n = 53) and the severity were shown above; with Vertigo/Nausea and vomitting as most frequent.

Figure 1, below illustrates the various vestibular disorders reported by the participants and the various degrees of severity. Among these, Vertigo; nausea and vomiting were most common and were recorded in 24 (45%) and 21(40%) respectively, of the participants.

**Figure 2:**
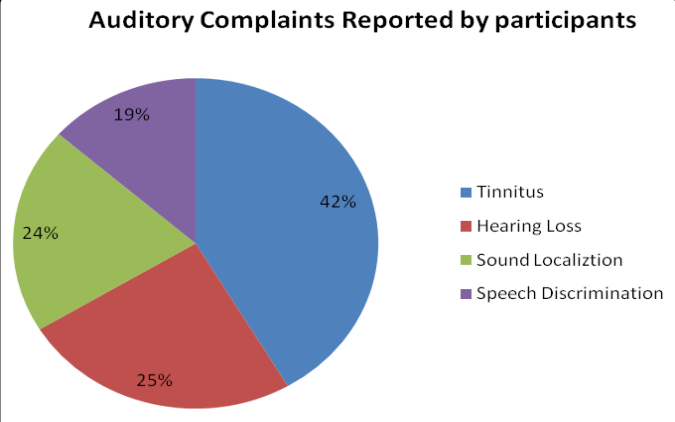
**Frequent auditory complaints reported by the participants (n = 53)**. Tinnitus and hearing loss were most preponderant auditory features recorded. It is worthy of note that the total % here is 110% because the symptoms are not mutually exclusive and therefore some occur in combinations.

Tinnitus 23(42%) was the most frequently reported auditory complaint reported by the participants. Hearing loss of different degrees was the second most common 13(25%), out of which 21% reported severe hearing losses in high frequencies. The frequency of distribution and reported severity of other auditory symptoms were as illustrated in Figure 2 below.

Objective audiological assessment was completed in 49(92%), 4 had a clash with their time for medication and could not complete the investigations. From the aforementioned 23(47%) had objective confirmed bilateral hearing losses of varying degrees (mild to profound) as shown in Table 1 below compared with 25% recorded subjectively from patients (Figure 2).

**Table 1:** Audiometric data for the participants (n = 49)

Degree of Hearing loss (dB HL)	N	Percentage
Normal (PTA: -10 to 25)	26	53%
Mild (PTA: 26 to 40)	7	14.5%
Moderate (PTA: 41 to 55)	7	14.5%
Moderately severe (PTA: 56 to 70)	3	3%
Severe (PTA: above 71)	6	12%
Total	49	100

*ALL participants had bilateral Sensorineural Hearing Loss of varying degrees as shown in the table. The frequencies of hearing losses were mainly beyond 4000 kz.

PTA--Pure Tone Audiogram; dB HL--Decibel Hearing Loss

Statistical calculations using Z-test 2 sample showed no statistical difference in the frequency of hearing losses recorded among the MDR and XTR TB groups 20/42 Vs 3/8; Z = 0.46 and P = 0.64. This suggests that the drug rather than the severity of disease was main determinant of level of hearing loss.

Further analysis of the results using inferential statistics (Chi-square, χ^2^) revealed no association between the gender of the participant and hearing loss (PTA) (P = 0.16, ά = 0.05). There was also no significant correlation between the age of the participants and the degree of hearing loss (p = 0.13, ά = 0.05).

## Discussions

The side effects emanating from the amino glycosides used in the MDR and XTR appears a potential threat to the successful management of this recalcitrant illness. The above findings of 42% cases of tinnitus and 25% reporting hearing losses among the in-patients, who were supposedly under the surveillance of the physician is rather a major health issue. The audiometric findings even revealed a higher proportion (47%) of patients with significant hearing losses. This report is in consonance with the findings of Torun et al [4] via a retrospective study conducted in Turkey where Ototoxicity from MDR-TB therapy yielded 41.8%. Other reports include de Jagger et al [5], in a 14 day case controlled study carried out among patients on prolonged therapy of aminoglycosides for TB therapy in the Netherlands 18%; Schacht [6], 10-20% in acute infection therapy and more in tuberculosis.

Various reports from different parts of the world have shown wide variations in the incidence of ototoxicity but the fact remains that it is a global problem. However, these variations could be explained following the presence of inherent modifiers such as genetic predisposition to aminoglycoside. Growing evidence has shown that mitochondrial 125rRNA gene mutations leading to 12S rRNA A1555G were under lying factors to aminoglycoside ototoxicity susceptibility particularly in Asian populations [7] Many families were found to transmit the aminoglycoside gene via matrilineal inheritance. The mitochondrial ribosome of the cochlear hair cells are believed to be the target attacked by the amino glycosides leading to processing of abnormal DNA or interference in the translation process culminating in an irreversible auditory malfunction [7,8].

A recent research finding has suggested that this genetic predisposition could be a significant factor among the South Africans [9] A genetic study carried out by the above worker and team, mapped genes A1555G and A827G among the Black race and Afrikaners of South Africa respectively, thus making them susceptible to aminoglycoside ototoxicity. This may justify the high incidence of oto-toxicity recorded in our study since the drugs were administered in the correct dosage by the managing physicians.

It is worthy of note that there was no significant correlation between sex, age or severity of illness (MDR or XDR TB) and ototoxicity pattern in this study. This is in agreement with an earlier study by de Jagger, et al [5] and strongly suggests that the ototoxic effect solely arises from the therapy. In addition there was no statistical correlation between the period of therapy and severity of hearing loss/other auditory symptomatologies. Thus, a little exposure could lead to severe auditory dysfunctions depending on individuals' genetic make-up. This is why it might be very difficult to define a generally acceptable duration for treatment of MDR and XDR TB patients.

Quite significant number of respondents reported vestibular symptoms other than nausea and vomiting. Vertigo (45%) and other related spatial disorders such as sensation of falling (40%) appear to be prominent features of the vestibulotoxicity from the anti MDR, TDR drugs. In a related report by WHO TB control sites, 14.8% had prominent vestibular symptoms against 12% hearing losses [10] although this is contrary to our report whereby the percentage hearing losses were more, however; it emphasises the importance of balance disturbances. Most often, less attention is paid to the vestibulotoxic symptoms of the ototoxic agents used in the Resistant TB therapy. However; the adverse complications following severe vestibular disturbances like falls and fractures (especially among the elderly), psychiatric disturbances, increase suicidal tendencies and generally poor quality of life are well documented [11-14]

## Limitations of the study

It would have been ideal to perform objective pre and post treatment audiological studies on all the patients using Ultra high frequency audiometry; the latter monitors frequencies above 8 kHz which aminoglycoside ototoxicity also produces. The monitoring of the serum concentrations for aminoglycosides through-out the course of treatment to regulate and also determine toxic levels would have been desirable; however the logistics for the research work did not support these.

## Conclusions

From the above findings it is pertinent that physicians and health care givers need to do more than present. A multi disciplinary surveillance of the patients diagnosed for MDR and XDR prior, during and after the completion of recommended regimen is imperative.

Again we posit that, such patients require monitoring of serum concentrations for aminoglycosides; genetic screening when possible, for the rRNA and related defective genes for aminoglycoside ototoxicity prior to commencement of therapy. This will aid informed consent and guarded treatment. During the course of therapy, monitoring should go beyond ward admission; the Audiologist, Otolaryngologist, Nephrologists and Hepatologists should be involved to ensure the protection and rehabilitation of vulnerable organs.

Finally, a permanent solution appears to lie on ability to discover a safer and effective chemotherapy for the treatment of MDR and XDR TB. Therefore, Pharmacologists and Chemists must increase their experimental takes toward finding an alternative and complete replacement for aminoglycosides in this respect.

## Competing interests

The authors declare that they have no competing interests.

## Authors' contributions

**LR **conceptualized the research topic and participated in the data collection and writing of the manuscript whereas **TSI **performed the literature search, writing of manuscript and final review of the manuscript. All authors read and approved the final manuscript.

## Authors' information

**LR **is Lecturer and Researcher in the Division of Communication Sciences & Disorders, Faculty of Health Sciences, University of Cape Town Groote Schuur Hospital, Old main building, F-45, Observatory, Cape Town, 7925, South Africa. **TSI**, is a Senior Lecturer Department of ENT Surgery, College of Health Sciences, University of Abuja, PMB 117 Abuja Nigeria; and Association of African Universities(AAU) Scholar/Visiting Lecturer to Division of Communication Sciences & Disorders, Faculty of Health Sciences, University of Cape Town

## Supplementary Material

Additional file 1 **Contains the proforma and some essential questions represented in the questionnaiore administered to all patients**. Click here for file
