# Supplementary material for: Cochleo-vestibular clinical findings among drug resistant Tuberculosis Patients on therapy-a pilot study
Source: Int Arch Med. 2012 Jan 31;5:3. doi: 10.1186/1755-7682-5-3 (PMC3284867; doi:10.1186/1755-7682-5-3)
Supplement: Additional file 1 — Contains the proforma and some essential questions represented in the questionnaiore administered to all patients. [file 1755-7682-5-3-S1.DOC]

**Additional file 1**

**Self-reported auditory dysfunction, participation restrictions and activity limitations questionnaire**

Date: Participant number:

**Demographic Information**

Name (Optional):

Sex □ Female □ Male

Date Birth (dd/mm/yy):

Address (optional):

Years of Formal Education:

**Current Marital Status:**

1. Never married □ 4. Divorced □
2. Currently married □ 5. Widowed □
3. Separated □ 6. Cohabitating □

**Current occupation:**

1. Paid employment □ 6. Retired □
2. Self employment □ 7. Unemployed (health reason) □
3. Non-paid work (volunteer, charity) □ 8. Unemployed (other reason) □
4. Student □ 9 Other □

Medical Diagnosis of existing Main Health Conditions **[Please NOTE whether the patient is MDR or XDR TB Patient:**

1. No medical condition exists
2. ................................
3. ................................
4. A health condition (disease, disorder, injury) exists, however its nature or diagnosis is not known.

**BRIEF HEALTH INFORMATION**

□ **Self Report** □ **Clinician Administered**

**X.1** How do you rate your physical health in the past month?

Very good □ Good □ Moderate □ Bad □ Very bad □

**X.2** How do you rate your mental and emotional health in the past month?

Very good □ Good □ Moderate □ Bad □ Very bad □

**X.3** Have you been hospitalized in the last year?

- NO □ YES

*If YES, please specify reason(s) and for how long?*

1. _____________________________; ___. ___. ___ days

2. _____________________________; ___. ___. ___ days

**X.4** Are you taking any medication (either prescribed or over the counter)?

- NO □ YES
- *If YES, please specify major medications*

1. _____________________________ 2. _____________________________

**X.5** Do you smoke?

- NO □ YES

**X.6** Do you consume alcohol or drugs?

- NO □ YES

*If YES, please specify average daily quantity*

Tobacco: __________________________

Alcohol: __________________________

Drugs: __________________________

**X.7** Do you use any assistive device such as glasses, hearing aid, wheelchair, etc.?

- NO □ YES

*If YES, please specify:* _________________________________­­­­­­­_____________

**X.8** IN THE PAST MONTH, have you cut back (i.e. reduced) your usual activities or work because of your *health condition*? (disease; injury; emotional reasons; alcohol; drug use)

- NO □ YES

If yes, how many days? _____

**Other questions:**

**X. 9** Has anyone ever told you that some TB medications may affect your hearing?

- NO □ YES

**X10**. Have you ever worked in a noisy environment?

- NO □ YES

If yes, please specify:_________________________________________

**Impairment of body functions**

Keys:

0- No impairment; 1- Mild impairment; 2- Severe impairment

| **SENSORY FUNCTIONS AND PAIN** | **0** | **1** | **2** |
| --- | --- | --- | --- |
| *Do you have problems hearing sound and speech around you?* |  |  |  |
| *Do you have problems hearing the difference between different sounds and people’s voices?* |  |  |  |
| *Do you have problems telling where the sound is coming from?* |  |  |  |
| *Do you have problems telling the difference between speech sounds and other sounds?* |  |  |  |
| *Do you have any other problems with your hearing that I did not ask you about?* |  |  |  |
| *Are you able to reach up for items or bend down whilst maintaining your balance?*  *( e.g. reaching for something on a high shelf)* |  |  |  |
| *Are you able to walk steadily on uneven turf or in the dark without losing balance?* |  |  |  |
| *If you roll over in bed do you feel dizzy, like you will fall over?* |  |  |  |
| *Do you ever hear ringing sound in your ears?* ***If answered “YES” How often do you ring sounds in your ear(s)? Is it in one or both ears? Are you currently receiving help for the ringing in your ear(s)?*** |  |  |  |
| *Do you ever experience a sensation of motion (either yourself or your environment), sensation of rotating, swaying or tilting?* |  |  |  |
| *Do you sometimes feel as though you are about to fall?* |  |  |  |
| *Do you ever feel nauseas when you are dizzy?* |  |  |  |
| *Do you ever feel any pain or itchiness in your ear?* |  |  |  |
| *Do your ears ever feel blocked or full?* |  |  |  |
| *Do you ever experience any of the following symptoms?*  *cold sweats, oscillopsia, nausea, vomiting, headaches, ataxia, lightheadedness* |  |  |  |

| **COMMUNICATION** |  |  |
| --- | --- | --- |
| *Do you have any problems in understanding spoken words?* |  |  |
| *Are you able to pick up body language?* |  |  |
| *Do you speak? Do you tell stories well? (making words, sentences)* |  |  |
| *Do you Use gestures & symbols to convey messages, e.g. shaking head to indicate disagreement or nodding head to indicate agreement?* |  |  |
| *Do you have any difficulties with having a conversation with one person?* |  |  |
| *Do you have any difficulties with having a conversation with more than one person?* |  |  |
| *Do you have any difficulties discussing a specific topic with one person?* |  |  |
| *Do you have any difficulties discussing a specific topic with more than one person e.g. elections?* |  |  |
| *Do you have any difficulties using for example telephone, cellphones?* |  |  |
| *Do you look at peoples lips when they speak, do you have to be facing them?* |  |  |
